# Predicting Residential Exposure to Phthalate Plasticizer Emitted from Vinyl Flooring: Sensitivity, Uncertainty, and Implications for Biomonitoring

**DOI:** 10.1289/ehp.0900559

**Published:** 2009-10-16

**Authors:** Ying Xu, Elaine A. Cohen Hubal, John C. Little

**Affiliations:** 1 Department of Civil, Architectural and Environmental Engineering, University of Texas at Austin, Austin, Texas, USA; 2 National Center for Computational Toxicology, U.S. Environmental Protection Agency, Research Triangle Park, North Carolina, USA; 3 Department of Civil and Environmental Engineering, Virginia Tech, Blacksburg, Virginia, USA

**Keywords:** biomonitoring, exposure, modeling, phthalates, plasticizers, semivolatile organic compounds, sensitivity, SVOCs, uncertainty

## Abstract

**Background:**

Because of the ubiquitous nature of phthalates in the environment and the potential for adverse human health effects, an urgent need exists to identify the most important sources and pathways of exposure.

**Objectives:**

Using emissions of di(2-ethylhexyl) phthalate (DEHP) from vinyl flooring (VF) as an illustrative example, we describe a fundamental approach that can be used to identify the important sources and pathways of exposure associated with phthalates in indoor material.

**Methods:**

We used a three-compartment model to estimate the emission rate of DEHP from VF and the evolving exposures via inhalation, dermal absorption, and oral ingestion of dust in a realistic indoor setting.

**Results:**

A sensitivity analysis indicates that the VF source characteristics (surface area and material-phase concentration of DEHP), as well as the external mass-transfer coefficient and ventilation rate, are important variables that influence the steady-state DEHP concentration and the resulting exposure. In addition, DEHP is sorbed by interior surfaces, and the associated surface area and surface/air partition coefficients strongly influence the time to steady state. The roughly 40-fold range in predicted exposure reveals the inherent difficulty in using biomonitoring to identify specific sources of exposure to phthalates in the general population.

**Conclusions:**

The relatively simple dependence on source and chemical-specific transport parameters suggests that the mechanistic modeling approach could be extended to predict exposures arising from other sources of phthalates as well as additional sources of other semivolatile organic compounds (SVOCs) such as biocides and flame retardants. This modeling approach could also provide a relatively inexpensive way to quantify exposure to many of the SVOCs used in indoor materials and consumer products.

Because of their substantial and widespread use, phthalates have become ubiquitous environmental contaminants (Koch et al. 2003; Weschler and Nazaroff 2008; Wormuth et al. 2006). More than 3.5 million tons of phthalates are used worldwide each year, primarily as plasticizers in flexible polyvinyl chloride (PVC) products (Cadogan and Howick 1996). Di(2-ethylhexyl) phthalate (DEHP) is an important phthalate, with more than two million tons produced globally each year (Lorz et al. 2002). About 90% of phthalates are found in numerous consumer products, including floor and wall coverings, car interior trim, clothing, gloves, footwear, wire insulation, artificial leather, and toys (Afshari et al. 2004; Bornehag et al. 2005; Müller et al. 2003). DEHP is mainly used in PVC products such as vinyl flooring (VF), where it is typically present at concentrations of about 20–40% (wt/wt) (Clausen et al. 2004; Deisinger et al. 1998). Because phthalate plasticizers are not chemically bound to the product materials, they are emitted slowly into the surrounding environment (Müller et al. 2003; Wormuth et al. 2006) and have become widely recognized as major indoor pollutants (Bornehag et al. 2005; Clausen et al. 2003; Fromme et al. 2004; Jaakkola and Knight 2008; Wensing et al. 2005; Weschler et al. 2008; Xu and Little 2006).

The ubiquitous human exposure to phthalates (Wormuth et al. 2006) is of concern because toxicologic studies in animals have demonstrated considerable adverse effects of phthalates and their metabolites (National Toxicology Program 2006). Because of the extensive environmental contamination with phthalates, a need exists to identify the most important sources and pathways of exposure [National Research Council (NRC) 2006]. Levels of phthalate metabolites measured in the general population using biomonitoring methods provide direct evidence of widespread human exposure (Calafat and McKee 2006; Centers for Disease Control and Prevention 2005; Heudorf et al. 2007). Biomonitoring data suggest that more than 75% of the U.S. population is exposed to phthalates (Silva et al. 2004). For phthalates with short alkyl chains, monoesters represent the major human metabolites, although in the case of DEHP, diisononyl phthalate, and diisodecyl phthalate, the monoesters are further metabolized. Exposure estimates based on urinary monoester concentrations might underestimate the population’s actual exposure to these specific phthalates (Wormuth et al. 2006). When urinary concentrations of secondary metabolites are measured, the estimate increases to 95% (Kato et al. 2004). Results of recent biomonitoring studies, in which phthalate metabolites were measured, are reviewed in Heudorf et al. (2007). Using mean body burden of DEHP expressed as urinary excretion of DEHP metabolites, they estimated that the effective intake of DEHP is higher in children than in adults and may occur at levels of significant concern. Data are not available for children < 3 years of age.

Interpretation of biomonitoring data for public health decision making requires contextual information to understand the potential for adverse health impacts and to identify effective interventions (Albertini et al. 2006; Bahadori et al. 2007). Just as additional information is required to relate a measured concentration of a chemical in a human tissue or fluid to the administered doses used in animal toxicity studies (Clewell et al. 2008), additional information is required to relate biomonitoring data to measures of the parent compound in environmental media (Fromme et al. 2007; Georgopoulos et al. 2008).

Although information on predominant sources, pathways, and routes of exposure is required to protect human health and the environment (NRC 2006), exposure to phthalates is difficult to evaluate because phthalates are so ubiquitous and because phthalate concentration measurements are hampered by contamination (Koch et al. 2003). To complicate matters, phthalates are sorbed strongly to surfaces, as do other semivolatile organic compounds (SVOCs) such as biocides and flame retardants (Weschler and Nazaroff 2008). A relatively small gas-phase concentration, such as 0.1 ppb, is sufficient for meaningful vapor transport of a phthalate ester and its consequent partitioning between the gas phase and indoor surfaces, including airborne particles and settled dust (Weschler 2003). Adibi et al. (2008) measured phthalate metabolite concentrations in urine samples from 246 pregnant women and correlated these with indoor air concentrations. They concluded that a single indoor air sample may be sufficient to characterize phthalate exposure in the home. In the recent Children’s Total Exposure to Persistent Pesticides and Other Persistent Organic Pollutants (CTEPP) study, the U.S. Environmental Protection Agency (U.S. EPA; 2005) measured concentrations of > 50 target compounds in multimedia samples from the homes and daycare centers of 260 preschool-age children. The two phthalates targeted in the CTEPP study were detected in residential air and house dust and on various interior surfaces and dermal wipes. The measured phthalate concentrations were among the highest of any of the target compounds, including pesticides, polycyclic aromatic hydrocarbons, and polychlorinated biphenyls. Based on an analysis of data from the CTEPP study, Xu et al. (2009) developed a model to predict emission and transport of DEHP and to estimate the potential exposure through different pathways.

Using DEHP in VF as an illustrative example, we extended the Xu et al. (2009) model to predict DEHP emissions and potential exposures via inhalation, dermal absorption, and oral ingestion of dust after the installation of VF in a family residence. Rather than conduct an exhaustive exposure assessment, we illustrate an approach that can be used to identify the important sources and pathways of exposure associated with phthalates in indoor materials and consumer products. As a result, we conducted sensitivity and uncertainty analyses to identify which model parameters have the greatest influence on exposure and to show why biomonitoring alone cannot easily be used to identify individual sources of exposure in the general population. Finally, we briefly discuss how the modeling approach could be generalized to include other sources of SVOCs, as well as emissions, transport, and exposure in other environmental media.

## Model Description and Results

**Figure 1:**
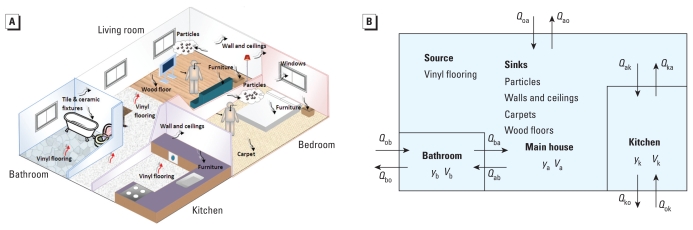
Schematic representation of the three-compartment residential model showing DEHP emitting from the VF sources and sorbing to the various sinks, including walls, ceilings, carpets, wood floors, and suspended particles with arrows and double subscripts on *Q* indicating direction of air flow. Abbreviations: a, main house; b, bathroom; k, kitchen; o, outside; *Q*, air flow; *V*, volume of compartment; *y*, gas-phase DEHP concentration.

As shown in Figure 1, DEHP is emitted from VF to the air in a typical residence that we divided into three compartments: kitchen, bathroom, and the main house. The emission rate is controlled by partitioning between the VF and the adjacent air, as well as the mass-transfer coefficient within the boundary layer above the VF. The gas-phase DEHP is sorbed on interior surfaces, including walls, ceiling, wood floor, carpet, furniture, windows, tile, ceramic fixtures, and particles through partitioning mechanisms. We obtained the infiltration/exfiltration rates and ventilation rates between rooms shown in Table 1 from measurements made by Wilkes et al. (1992) in a five-room house. We estimated the interior surface area of furnishing and materials using typical surface:volume ratios for American houses established by Hodgson et al. (2005) (Table 1). VF comes in two main types. The one used in homes is softer and has a higher phthalate content than the more rigid one used in commercial applications. For modeling purposes, we use the commercial type because the emission characteristics and DEHP content have been comprehensively investigated in previous studies (Clausen et al. 2004; Xu and Little 2006; Xu et al. 2008, 2009).

We obtained sorption isotherms for phthalates on different interior surfaces from data collected in a residential field study and a laboratory chamber study (Xu et al. 2009). In the CTEPP field study (U.S. EPA 2005), 48-hr integrated samples were collected simultaneously from children’s daycare centers and from their homes in either North Carolina or Ohio. The samples were collected from residential air, house dust, interior surfaces, and dermal wipes. Clausen et al. (2004) conducted laboratory experiments to study DEHP uptake by dust on PVC flooring in a chamber for laboratory investigations of materials, pollution, and air quality (CLIMPAQ). We used the DEHP concentrations in the dust and gas phase to determine the DEHP partition coefficient between dust and air. Log-linear relationships between equilibrium parameters and chemical vapor pressure were obtained, and the partition coefficients for DEHP on different interior surfaces calculated based on the vapor pressure of DEHP (Xu et al. 2009). We estimated the value of the mass-transfer coefficient for the boundary layer adjacent to the various surfaces using correlation equations (Axley 1991).

**Figure 2:**
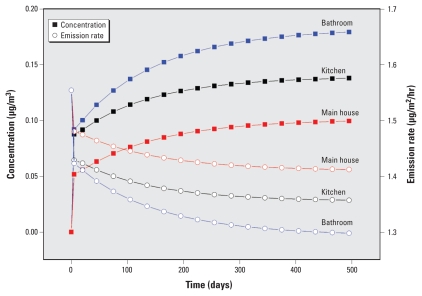
Change over time in emission rate and gas-phase concentration of DEHP emitted from VF.

The model was used to estimate DEHP emission and transport after VF was installed in a residence (Figure 2). The three compartments reached steady state within about 1.5 years. The steep initial rise in DEHP concentration occurred because the rate at which it is emitted from the VF is initially faster than the rate at which it is taken up by the interior surface sinks. Compared with the other two compartments, the main house had the lowest gas-phase concentration because of the larger ratio of sorption surface area (e.g., carpet and furniture) to emission surface area. The lower the gas-phase concentration, the higher the concentration gradient in the boundary layer above the VF and the higher the emission rate. As shown in Table 2, the predicted steady-state concentrations are similar to those measured in homes in the United States and Europe.

Based on these results, we evaluated exposures to gas-phase DEHP in air, particle-bound DEHP in air, and DEHP in settled dust. The exposure pathways of interest were inhalation of vapor, inhalation of particles, dermal sorption of DEHP, and oral ingestion of household dust. Both children and adults were considered in the assessment. We quantified the magnitude, frequency, duration, and time pattern of contact with DEHP using the screening-level assessment described by Xu et al. (2009).

**Figure 3:**
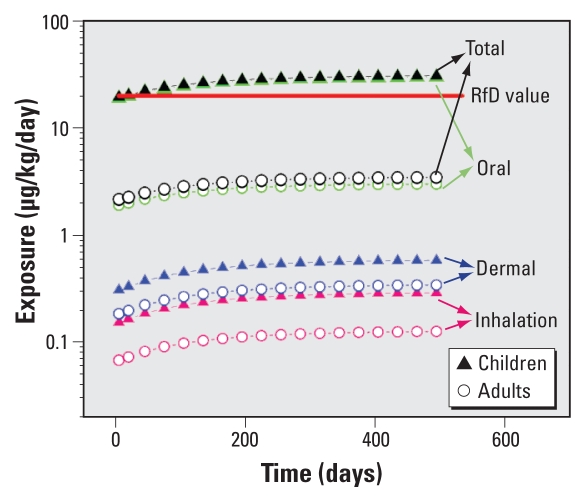
Change in time in predicted exposure to DEHP emitted from VF through inhalation, dermal sorption, and oral ingestion of dust. Total represents the sum of inhalation, dermal, and oral exposures, and the arrows indicate exposure values associated with various expsoure routes.

Figure 3 shows the change in time in exposure for adults and children (between the first and third year of life) through inhalation, dermal sorption, and oral ingestion of dust. Exposure reaches a steady level after about 1.5 years. Children experience 2–10 times greater exposure than do adults. The results are similar to those of Heudorf et al. (2007) who modeled ambient exposure data and concluded that children may be more highly exposed than adults. The reference dose (RfD) is 20 μg/kg/day according to the U.S. EPA. For children, exposure through oral intake via dust is two times higher than the RfD, although the assumed dust intake rate of 10.3 mg/kg/day may be high (Xu et al. 2009). For DEHP, the primary route of exposure is oral ingestion of dust; inhalation and dermal sorption do not appear to be dominant exposure pathways, which is consistent with observations of Clark et al. (2003).

### Sensitivity analysis

We conducted a sensitivity analysis to identify the critical model variables for total exposure and for each exposure pathway. Here, we computed exposure after each of the three compartments had reached steady state. The sensitivity of the model variables were assessed by computing the percent change in exposure per unit increase in an input variable. The baseline conditions are those used for the results shown in Figure 3. Table 3 shows the results of the sensitivity analysis, along with the baseline values of selected model variables. Sensitivity to all model parameters is provided as Supplemental Material (doi:10.1289/ehp.0900559.S1 via http://dx.doi.org/).

The properties affecting the source strength (initial DEHP concentration in VF, partition coefficient between VF and air, and surface area of VF) have a significant effect on all the exposure pathways. Increasing the mass-transfer coefficient (*h*_m_) will increase the emission rate and significantly increase exposure, whereas increasing the ventilation rate will reduce exposure. Note, however, that the latter assumes an increase in air-exchange rate alone, without increasing the mass-transfer coefficients, which would tend to increase as ventilation increases. Increasing either the total suspended particle (TSP) concentration or the particle/air partition coefficient total total suspended particle (TSP) concentration or the particle/air partition coefficient (*K*_particle/air_) is equivalent, either of which has a stronger impact on dermal sorption and oral ingestion than on inhalation. The reason is that increasing sorption on particles reduces the gas-phase concentration, and both dermal sorption and oral ingestion decrease significantly. However, because particles contribute 80% of the inhalation exposure, the two effects were cancelled, and inhalation exposure increased only slightly. As expected, exposure duration and body weight also strongly influenced the resulting exposure.

### Uncertainty analysis

Model variables can be defined in terms of a probability distribution function (PDF) that is derived from a limited set of observations. We adopted a simple Monte Carlo analysis to account for uncertainty associated with the model parameters, as well as natural variability. A PDF for each of the important variables identified in the sensitivity analysis was randomly sampled to obtain a value for the variable. This set of model variables was then used to calculate exposure. The uncertainty analysis consisted of 1,000 such exposure computations, which we used to derive a cumulative distribution function describing an estimate of the uncertainty in exposure.

**Figure 4:**
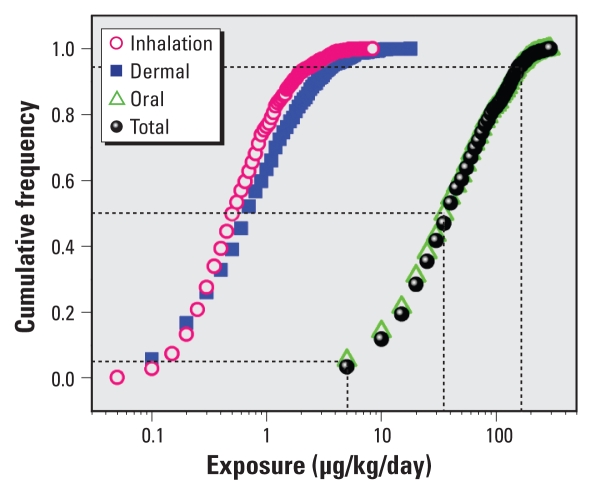
Uncertainty in predicted steady-state exposure to DEHP emitted from VF via inhalation, dermal sorption, and oral ingestion of dust.

As shown in Table 4, we developed ranges in selected model parameters from data presented in other studies or obtained directly from the literature. We used simple uniform distributions because of the relative paucity of data, even though this may overestimate uncertainty. Figure 4 summarizes the uncertainty for the individual exposure pathways as well as for total exposure. Overall, exposure varies from about 5 μg/kg/day at the 5th percentile to about 180 μg/kg/day at the 95th percentile, a roughly 40-fold difference. The median value (50th percentile) of about 38 μg/kg/day is almost double the RfD.

## Discussion

The high surface concentrations of phthalate on human skin observed in the CTEPP study were generally assumed to have been the result of dermal transfer. Cohen Hubal et al. (2008) studied the dermal transfer of chemicals from contaminated surfaces (e.g., floors and furniture) to skin, providing a range of measured transfer efficiencies, all of which were < 100%. Closer examination of the CTEPP data shows that the measured concentrations on skin were almost always higher than the measured concentrations on other surfaces. To investigate if the high dermal loadings are caused by transfer of chemicals from contaminated surfaces or from partitioning with air, we conducted a multilinear regression. As shown in Table 5, the skin concentrations are strongly correlated with the concentrations in air and are not correlated with the hard surface concentrations. Because equilibrium is established fairly quickly between surfaces and air, the dermal transfer of phthalate from surface to skin may not have a substantial influence on exposure (Xu et al. 2009). This is supported by the recent results of Adibi et al. (2008), who concluded that a single indoor air sample may be sufficient to characterize phthalate exposure in the home.

In the simple sensitivity analysis described above, we varied only one parameter at a time. However, when the ventilation rate is increased, the mass-transfer coefficients will also increase because of the higher air velocity near the surfaces. As a result, the emission rate of DEHP from VF will be higher and the rate of DEHP sorption to interior surfaces will be faster. Thus, the predicted exposure will decrease by only 25% compared with the decrease of 46% predicted in the simple sensitivity analysis. In addition, the boundary layer of air adjacent to the skin will be thinner and the mass-transfer resistance will be reduced. Because the external gas-phase resistance controls the overall rate of dermal permeation (Xu et al. 2009), the permeability of DEHP through the skin will be enhanced, meaning that dermal exposure will actually increase by 13%, as opposed to the decrease of 46% found in the simple sensitivity analysis. This rather surprising result suggests that the use of indoor fans could increase the permeation rate of DEHP through the skin.

Many other interior surfaces, including clothing, bedding, rugs, newspapers, books, magazines, human hair, crockery, and cutlery have not been taken into account in our exposure model. To get a rough idea of the effect of including these additional surfaces, we nominally increased all interior surface areas by a factor of 3 from the model baseline conditions. In this case, sorption of DEHP to the much higher surface area doubles the time to reach steady state. Direct dermal sorption and ingestion from these other surfaces may increase the risk of DEHP exposure significantly. For example, DEHP would be expected to accumulate in clothes hanging in a cupboard. When these are worn, dermal sorption could increase substantially. De Coensel et al. (2008) studied the chemical contamination of clothes because of their direct or indirect exposure to moth repellent agents, which are similar to SVOCs, and concluded that clothes sorb high concentrations of contaminants, and that they should be considered as secondary sources of indoor air pollution. Although the surface/air partition coefficient for the interior surfaces did not have an effect on the predicted steady-state exposure, it will influence the time to reach steady state. The stronger the partitioning between interior surfaces and air, the longer it will take to reach steady state. For instance, doubling the wall and ceiling/air partition coefficient increases the time to steady state by about 50%.

Other sources, such as food packaging, may be important DEHP exposure pathways (Koch et al. 2003), and young children can be exposed by mouthing soft PVC toys and teethers (Petersen and Breindahl 2000). In addition, plasticized PVC is the most widely used electrical insulation material on wires and cables, with an estimated length of about 16 million kilometers in U.S. buildings today (Wilson 2009). By varying the DEHP content, cable manufacturers are able to produce wiring that remains flexible at low temperatures. These additional sources will result in higher DEHP concentrations in room air and dust and on skin. Many other sources of phthalates also exist in the environment. Because the model employs a mechanistic approach to predict exposure to DEHP emitted from VF, it should be relatively simple to generalize the model to include phthalates emitted from these other sources. As shown in the sensitivity analysis, the most influential, chemical-specific model parameters are the various mass-transfer and partition coefficients. The partition coefficients generally correlate well with vapor pressure, whereas the chemical-specific dependence of the mass-transfer coefficients is easy to estimate (Xu et al. 2009).

### Implications for biomonitoring

The ability to measure chemicals in humans (biomonitoring) is far outpacing the ability to reliably interpret these data for public health purposes, which has created a major knowledge gap (Bahadori et al. 2007). As discussed in the introduction, the use of biomonitoring data to design and evaluate public health interventions for compounds such as phthalates requires additional information on potential sources, temporal and spatial patterns of exposure, and a mechanistic understanding of the source-to-outcome continuum. The sensitivity and uncertainty analyses presented above suggest that a single phthalate (DEHP) in a single material (VF) could result in a population exposure that varies by as much as a factor of 40. This wide range in exposure would confound the interpretation of cross-sectional biomonitoring results.

In the context of human health risks, Calafat and McKee (2006) outline research needs for using DEHP biomonitoring data to inform exposure assessment. Their recommendations include the need to identify vulnerable segments of the population that may be more highly exposed to phthalates than is the general population and to identify sources of exposure to these vulnerable groups. The example we present in this article demonstrates the utility of physically based models for predicting concentrations of SVOCs as a function of time and space in residential environments. Such an approach combined with traditional scenario-based exposure algorithms facilitates identification of potentially vulnerable groups such as pregnant women, babies, and young children. Our example shows that the dependence on source and chemical-specific properties is relatively simple, suggesting that the model could be extended to include other sources of phthalates, as well as other characteristics of the indoor environment.

A recent report on phthalates and cumulative risk assessment by the National Academies (NRC 2008) recommends that the U.S. EPA should *a*) determine prenatal exposure to phthalates at relevant times during pregnancy; *b*) identify the most important sources of phthalate exposure in the general population; *c*) identify the full spectrum of phthalate metabolites, which are produced when phthalates enter the body, and identify the metabolites that can be used to reliably indicate phthalate exposure; *d*) understand the reasons for differences in susceptibility to phthalates based on age, species, and exposure route; and *e*) explore the potential of phthalates to cause synergisms in combination with other antiandrogens. It is clear that biomonitoring alone cannot provide answers to recommendations *b* and *d*. In contrast, the approach articulated in this article can be used to identify the most important sources of phthalate exposure and can explain differences in susceptibility to phthalates based on age, species, and exposure route. Although our example focuses on emissions from a specific source (VF) to a specific environmental medium (air), it can most likely be generalized to many other sources emitting various SVOCs (e.g., insulated wiring, cosmetics, personal-care products, pharmaceuticals, medical devices, children’s toys, food packaging, and cleaning and building materials) into a wide range of environmental media (air, food, water, saliva, and even blood), provided that appropriate behavioral and product use factors are incorporated. Assuming that the necessary model development, parameter identification, and model validation are undertaken, the approach could prove to be a relatively inexpensive and efficient way to identify potential exposures associated with many of the SVOCs used in indoor materials and consumer products.

## Figures and Tables

**Table 1:** Conditions for the three-compartment residential model.

Compartment	Main house	Kitchen	Bathroom
Volume (m^3^)	128	35	15
Flow rate (m^3^/hr)	*Q*_oa_ 65	*Q*_ok_ 12	*Q*_ob_ 1.1
	*Q*_ao_ 44	*Q*_ko_ 32	*Q*_bo_ 2.1
		*Q*_ak_ 44	*Q*_ab_ 14
		*Q*_ka_ 24	*Q*_ba_ 13
Surface area (m^2^)			
VF	19.2	14.4	6.20
Walls and ceilings	124	34.0	23.3
Carpet	35.8	—	—
Wood floor	32.0	—	—
Hard surface furniture	61.4	12.6	5.40
Windows and mirrors	5.12	1.75	1.05
Tile and ceramic fixtures	5.12	3.50	16.5
TSP (μg/m^3^)	20.0	20.0	20.0

Abbreviations: a, main house; b, bathroom; k, kitchen; o, outside.

**Table 2:** Predicted concentrations of DEHP in indoor air and household dust compared with those cited in the literature.

DEHP	Reference	*n*	Mean	Maximum	Present study
Gas-phase concentration (μg/m^3^)					0.1–0.18

	BAUCH (Beratung und Analyse—Verein für Umweltchemie) 1991	40	0.48	1.6	
	Sheldon et al. 1994	125	0.14	—	
	Rudel et al. 2003	102	0.07	1.0	
	Fromme et al. 2004	59	0.19	0.4	

Dust-phase concentration (μg/g)					2,000–3,500

	BAUCH 1991	12	950	3,100	
	Mattulat 2002	600	1,200	3,500	
	Rudel et al. 2003	101	340	7,700	
	Fromme et al. 2004	30	780	1,800	
	Weschler et al. 2008	30	776	1,542	

**Table 3:** Sensitivity of predicted steady-state exposure to selected model parameters.

Variable	Baseline value	Exposure pathway			
		Inhalation	Dermal	Oral	Total
DEHP concentration in VF (*C*_0_, μg/m^3^)	2.55 × 10^11^	1.00	1.00	1.00	1.00
Partition coefficient (*K*_vinyl/air_)	2.3 × 10^11^	−0.50	−0.50	−0.50	−0.50
Mass-transfer coefficient for flat surfaces (*h*_m_, cm/sec)	0.1	0.82	0.82	0.79	0.79
TSP concentration (μg/m^3^)	20	0.07	−0.42	−0.41	−0.41
Partition coefficient (*K*_particle/air_, m^3^/μg)	0.25	0.07	−0.42	−0.41	−0.41
Partition coefficient (*K*_dust/air_, m^3^/g)	21,100	0.00	0.00	1.00	0.97
Inhalation rate (IR, m^3^/day)	6.8	1.00	0.00	0.00	0.01
Exposure duration in main house (ED_3_, hr/day)	16.5	0.88	0.88	—	—
Skin surface area (SA, m^2^)	0.59	0.00	1.00	0.00	0.02
Overall skin permeability coefficient (*P*, cm/hr)	580	0.00	1.00	0.00	0.02
Daily intake rate of dust (DIR, mg/kg/day)	10.3	0.00	0.00	1.00	0.97
Body weight (kg)	11	−0.50	−0.50	—	—
Air exchange rate for three compartments (1/hr)	0.5	−0.46	−0.46	−0.46	−0.46
VF area in kitchen (*A*_1vinyl_, m^2^)	14.4	0.18	0.18	~0.22	~0.22
VF area in bathroom (*A*_2vinyl_, m^2^)	6.2	0.13	0.13	~0.25	~0.25
VF area in main house (*A*_3vinyl_, m^2^)	19.2	0.52	0.52	~0.34	~0.35

Sensitivity to all model parameters is provided in the Supplemental Material (doi:10.1289/ehp.0900559.S1).

**Table 4:** Parameter ranges used in uncertainty analysis.

Variable	Minimum	Maximum	References
Initial DEHP concentration in VF (*C*_0_, μg/m^3^)	2.25 × 10^11^	6.0 × 10^11^	[Bibr b12-ehp-118-253][Bibr b19-ehp-118-253]
Partition coefficient (*K*_vinyl/air_)	2.05 × 10^11^	5.45 × 10^11^	—
Mass-transfer coefficient for flat surfaces (*h*_m_, cm/sec)	0.03	0.29	Huang et al. 2004, Lin et al. 2004
TSP concentration (μg/m^3^)	12	66	Weschler et al. 2008
Partition coefficient (*K*_particle/air_, m^3^/μg)	0.22	0.28	Naumova et al. 2003
Partition coefficient (*K*_dust/air_, m^3^/g)	2,000	4 × 10^4^	Rudel et al. 2003, Weschler et al. 2008
Inhalation rate (IR, m^3^/day)	5	14.5	Paustenbach 2000
Exposure duration in main house (ED_3_, hr/day)	12.6	18.1	Cohen Hubal et al. 2000
Skin surface area (SA, m^2^)	0.59	1.7	U.S. EPA 1997
Overall skin permeability coefficient (*P*, cm/hr)	56	1,035	De Dear et al. 1997
Daily intake rate of dust (DIR, mg/kg/day)	1.03	10.3	Wensing et al. 2005
Body weight (kg)	9.15	62.2	U.S. EPA 1997
Air exchange rate for three compartments (1/hr)	0.1	1.1	Wallace et al. 2002
VF area in kitchen (*A*_1vinyl_, m^2^)	11.9	47.6	Hodgson et al. 2005
VF area in bathroom (*A*_2vinyl_, m^2^)	5.1	20.4	Hodgson et al. 2005
VF area in main house (*A*_3vinyl_, m^2^)	2.56	44.8	Hodgson et al. 2005

—, no references available.

**Table 5:** Multilinear regression to establish relationship between skin concentration^a^ and both air^b^ and hard surface^c^ concentrations.

Chemical/sample	*K*_1_ (m), slope for *x*_1_	*p*-Value for *K*_1_	*K*_2_, slope for *x*_2_	*p*-Value for *K*_2_
DBP				
Child hand wipe, *q*	58	0.027	0.07	0.89
Adult hand wipe, *q*	79	0.02	0.19	0.82
BBP				
Child hand wipe, *q*	140	0.045	0.03	0.26
Adult hand wipe, *q*	55	0.09	0.03	0.04
BPA				
Child hand wipe, *q*	1600	0.005	0.22	0.73
Adult hand wipe, *q*	950	0.004	0.31	0.37

Abbreviations: BBP, benzyl butyl phthalate; BPA, bisphenol A; DBP, dibutyl phthalate.

a (*q* in μg/m^2^),

b (*x*_1_ in μg/m^3^), and

c (*x*_2_ in μg/m^2^), where *q* = *K*_1_(*x*_1_) + *K*_2_(*x*_2_).
